# Acute aortic occlusion after microendoscopic laminectomy in a patient with lumbar spinal stenosis: A case report

**DOI:** 10.1097/MD.0000000000028347

**Published:** 2021-12-23

**Authors:** Hirokazu Inoue, Akira Sugaya, Yuya Kimura, Yasuyuki Shiraishi, Ryo Sugawara, Atsushi Kimura, Katsushi Takeshita

**Affiliations:** a Department of Orthopaedics, Jichi Medical University, 3311-1 Yakushiji, Shimotsuke, Tochigi, Japan; b Department of Cardiovascular Surgery, Jichi Medical University, 3311-1 Yakushiji, Shimotsuke, Tochigi, Japan.

**Keywords:** acute aortic occlusion, lumbar spinal stenosis, microendoscopic laminectomy, paralysis, plasminogen activator inhibitor type-1

## Abstract

**Rationale:**

Acute aortic occlusion is an uncommon disease with a high morbidity and high mortality. Clinical symptoms typically include acute lower limb pain, acute paralysis, and absent pulses. We report a very rare case of acute aortic occlusion causing complete paralysis of bilateral lower limbs following microendoscopic laminectomy.

**Patient concerns:**

A 64-year-old man with hypertension, hyperlipidemia, diabetes, and atrial fibrillation underwent microendoscopic laminectomy for lumbar spinal stenosis. After the operation, intermittent claudication improved significantly without neurological deficit. However, 7 days later, he developed complete paralysis of the bilateral lower limbs, extreme pain of the bilateral lower limbs, and mottling of the left extremity.

**Diagnosis:**

An emergency magnetic resonance imaging examination revealed no epidural hematoma behind the spinal cord, proscribing spinal cord compression. Computed tomography revealed occlusion of the infrarenal abdominal aorta. Blood tests revealed high values of total plasminogen activator inhibitor-1 before surgery.

**Interventions:**

The acute aortic occlusion was verified and underwent thrombectomy and right axillary-bifemoral bypass.

**Outcomes:**

Following the revascularization, the neurological deficit of the lower limbs improved. On follow-up after 1 year, the muscle strength of the bilateral lower limbs had returned to normal.

**Lessons:**

This case presentation highlights the necessity of early diagnosis and early revascularization. Moreover, a preoperative high value of plasminogen activator inhibitor-1 may indicate vascular complications including Acute Aortic Occlusion.

## 1 Introduction

Acute aortic occlusion (AAO) is a relatively rare disease with a high morbidity and high mortality. Previous studies report 30-day mortality rate of 19.9% to 52% in AAO, with complications that include bowel ischemia, paraplegia, and lower extremity ischemia.^[1–6]^ Grip et al demonstrated the incidence of AAO was 3.8 per 1 million person-years, with a mean age of 69.7 years.^[1]^ The most common comorbidities included cardiac disease, hypertension, and current smoker. Clinical signs in AAO typically involve acute lower extremity pain, absent pulses in the lower extremities, sensory disturbance, and pale skin.^[7–9]^ The symptoms can sometimes yield a mistaken diagnosis of epidural hematoma, cerebrovascular accident, or a central nervous system lesion.^[4,10]^ A delayed correct diagnosis can lead to increased morbidity and mortality. Ischemic complications including gastrointestinal malperfusion, renal infarction, and spinal cord infarction can then occur.

Postoperative spinal epidural hematoma is a rare complication after spinal surgery and can lead to acute paralysis during several hours or several days. The other causes of acute paralysis include spinal cord infarction, cerebral infarction, and AAO. To our knowledge, this is the first paper of AAO in patients with spinal disease after spine surgery. We present a case report of AAO in a patient with lumbar spinal stenosis after microendoscopic laminectomy.

## 2 Case presentation

A 64-year-old male was admitted with a history of right lower limb radiculopathy associated with neurogenic claudication of <50 m for 6 months. He had hypertension, hyperlipidemia, diabetes, and atrial fibrillation. The patient was on anti-coagulation medication (Warfarin 2.5 mg once daily) for atrial fibrillation. Lumbar magnetic resonance imaging showed spinal canal stenosis at L2/3 and L4/5. The patient underwent decompression with L2/3 and L4/5 microendoscopic laminectomy. Warfarin was stopped on preoperative day 5, while systemic heparin was started on the same day and was stopped 6 hours before surgery. On postoperative day 2, the patient walked free and the patient's right leg pain and claudication were improved. On postoperative day 3, warfarin was resumed.

**Figure 1:**
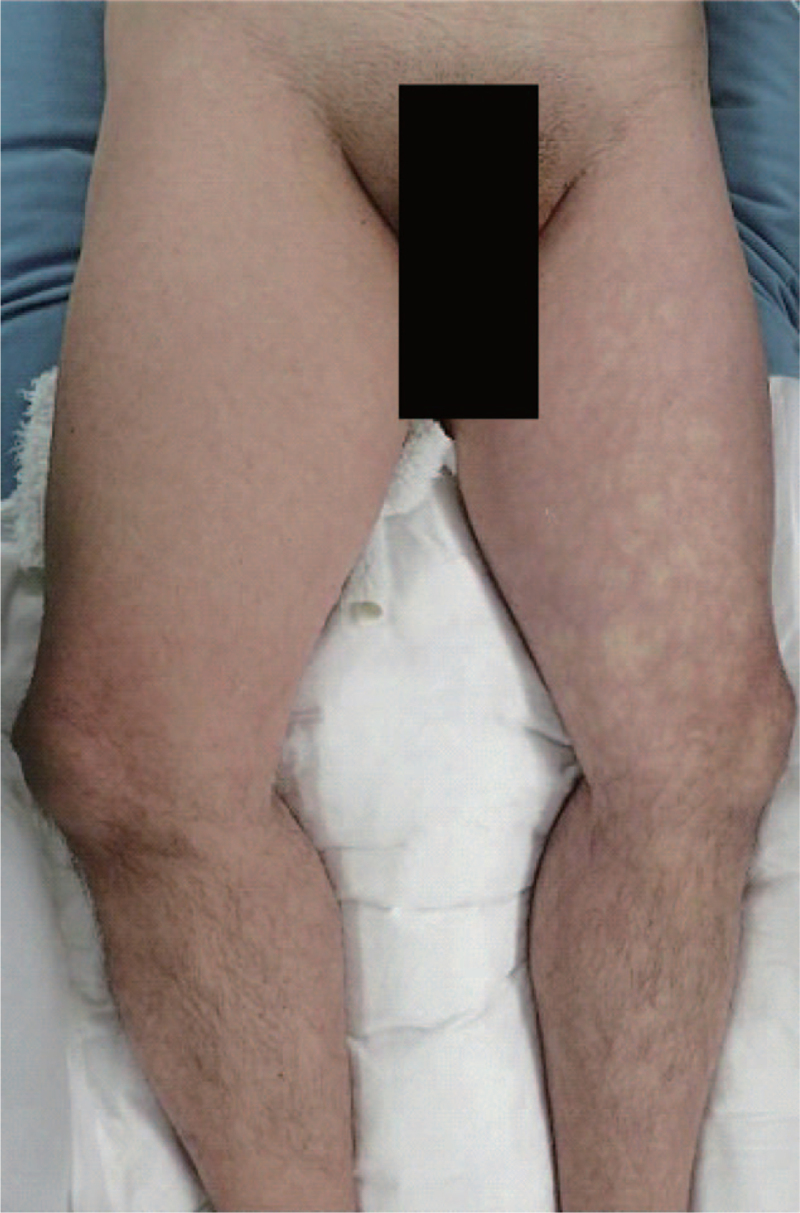
Photographs of the lower extremities. Preoperatively, the skin on the left thigh appears pale, mottled, and cyanotic, while ischemic changes of the skin on the right side are mild.

**Figure 2:**
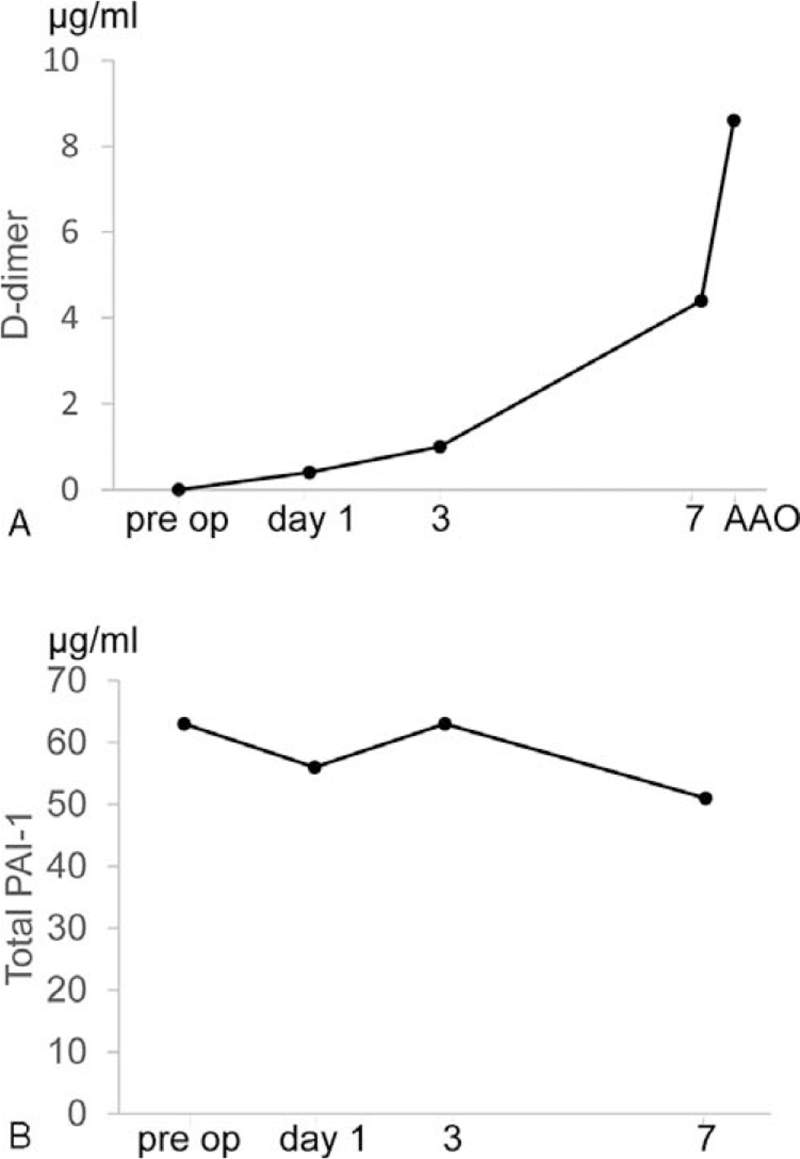
Changes in D-dimer, and total plasminogen activator inhibitor-1 (PAI-1). (A) Graph showing changes in D-dimer. D-dimer increased when acute aortic occlusion (AAO) occurred. (B) Graphs showing changes in total PAI-1. Total PAI-1 remained over 31 ng/ml perioperatively.

**Figure 3:**
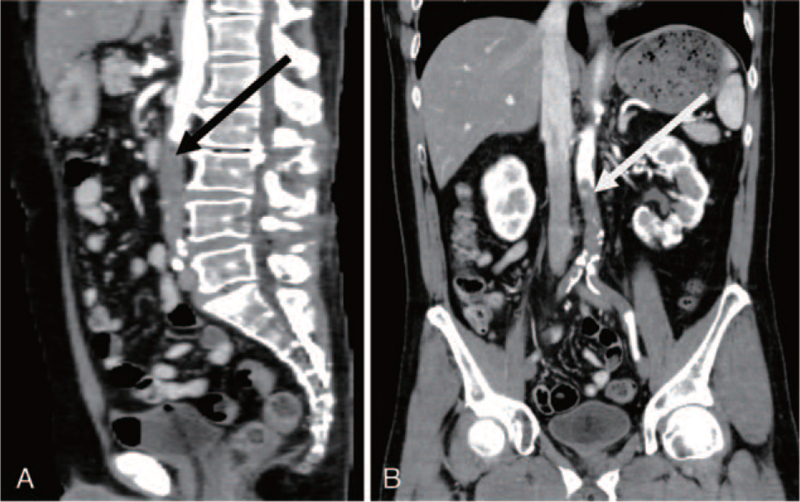
Visualized thrombus within abdominal aorta (arrow) on CT angiogram image of a patient with acute aortic occlusion. (A) Sagittal view of CT angiogram. (B) Coronal view of CT angiogram.

When the patient exhibited both strong leg pain and complete paralysis on postoperative day 7, his vital signs were as follows: temperature, 35.0°C; heart rate, 95 bpm; and blood pressure, 176/99 mm Hg. Physical examination revealed mottling of the left lower extremity and loss of the bilateral femoral pulses (Fig. 1). During the examination at AAO onset, it was evidenced that the mottling was progressing. Ischemic changes of the skin on the right lower extremity were mild. Blood tests revealed the following values: lactate dehydrogenase, 331 IU/L; creatine phosphokinase, 128 IU/L; serum creatinine level, 1.23 IU/L; and serum potassium ion concentration, 3.3 mEq/L. Figure 2 showed D-dimer and total plasminogen activator inhibitor-1 (PAI-1) from preoperation to postoperative day 7. D-dimer significantly increased after AAO, but high values of total PAI-1 continued after surgery. The test results for antithrombin III, protein C and S deficiencies, and anticardiolipin antibody were negative. The electrocardiogram showed atrial fibrillation. Magnetic resonance imaging showed no epidural hematoma behind the spinal cord, proscribing spinal cord compression. Computed tomography (CT) angiogram revealed occlusion of the infrarenal abdominal aorta and bilateral iliac arteries with reconstruction of flow via collaterals beginning at the bilateral common femoral arteries (Fig. 3).

The patient was immediately started on systemic heparin. The patient underwent thrombectomy and right axillary-bifemoral bypass. After revascularization, the paralysis of bilateral lower limbs improved. The patient did not develop myonephropathic metabolic syndrome or visceral ischemia. The postoperative course was uneventful, and additional surgical procedures were not required. The patient again received warfarin (2.5 mg once daily) after systemic heparin. At 1-year follow-up, he remained free of occlusion, paralysis, and intermittent claudication; but the numbness of the left leg extremity persisted.

## 3 Discussion

Postoperative paralysis is mainly the result of spinal epidural hematoma after spine surgery. But surgeons need to suspect other causalities such as spinal cord infarction, cerebral infarction, and AAO. AAO is infrequent. However, the diagnosis should not be difficult. Acute onset of bilateral leg pain, neurologic deficits, and lower extremity mottling should alert the physician to suspect AAO. Careful symptomology coupled with CT angiogram distinguishes neurologic from vascular causes and helps inform appropriate treatment (Fig. 3). Though the obvious diagnosis in most cases is performed, the results in patients with AAO continue to be marked with significant morbidity and mortality.

AAO may be associated with acute onset of neurologic disorder as a result of spinal cord ischemia from thrombotic or embolic etiology. Risk factors of embolism were heart disease and female gender, while risk factors of thrombosis were hypertension, tobacco smoking, and diabetes mellitus.^[3]^ Spinal cord infarction constitutes only 1% to 2% of all strokes and includes acute onset of paralysis, bowel and bladder dysfunction, and pain and temperature sensation. In many cases, proprioception and vibratory sensation are preserved.^[11]^ AAO usually occurs within the infrarenal aorta and can result from a thromboembolic event, direct thrombosis of an atherosclerotic aortic plaque, occlusion of an abdominal aortic aneurysm, or aortic dissection. This case had an infrarenal aortic occlusion including both strong leg pain and paralysis.

Survivors with AAO also develop major complications after treatment including: major complications of cardiovascular disorders (34%), major amputation (15%), acute kidney injury (57%) requiring haemodialysis (22%), and surgical site infection (6.2%). A second operation was required by 42% of patients.^[12]^ Grip et al described that most patients with AAO presented with bilateral acute limb ischemia (81.2%) and the amputation rate was 8.6%.^[1]^ In the present case, a good outcome was achieved despite the potentially lethal condition.

According to the clinical course, we believe the initial aortic occlusion occurred from an embolic event. Autolysis of clot through endothelium-medicated release of fibrinolytic agents, such as tissue-type plasminogen activator, is common.^[13]^ This mechanism has been demonstrated in the venous system after deep venous thrombosis and pulmonary embolism,^[14]^ but in the present case might have resulted from the arterial vasculature. In previous papers, elevated levels of PAI-1 were considered to be markers of thrombosis including venous thromboembolism (VTE).^[15–17]^ PAI-1 inhibits plasminogen activator which activates production of fibrin or thrombus.^[18]^ Platelets involve substantially elevated levels of PAI-1 compared to plasma, but 90% of platelet PAI-1 is inactive; whereas most PAI-1 in plasma is active.^[19]^ Watanabe et al demonstrated PAI-1 levels in patients with VTE increased higher than patients without VTE after pneumatic tourniquet release during total knee arthroplasty.^[20]^ In our previous study, preoperative PAI-1 in patients with DVT and VTE was significantly increased at a cut-off value of 31 ng/ml.^[21]^ We believe that high preoperative PAI-1 predicts thromboembolic events such as AAO.

## 4 Conclusion

The present case report showed that the abrupt onset of bilateral leg pain with neurologic deficits of paralysis, sensory disturbance, and mottled extremities, alerted the physician to AAO. Moreover, the preoperative high value of total PAI-1 propounds vascular complications including AAO. AAO continues to have high morbidity and mortality, and early diagnosis with rapid transfer for surgical intervention is essential for improving outcomes.

## Author contributions

**Conceptualization:** Hirokazu Inoue.

**Data curation:** Yuya Kimura, Yasuyuki Shiraishi.

**Investigation:** Akira Sugaya, Yuya Kimura.

**Project administration:** Hirokazu Inoue, Yasuyuki Shiraishi.

**Supervision:** Katsushi Takeshita.

**Validation:** Ryo Sugawara, Atsushi Kimura.

**Writing – original draft:** Hirokazu Inoue.

**Writing – review & editing:** Hirokazu Inoue.
